# Microscopy Study of (Ti,Nb)(C,N) Precipitation in Microalloyed Steels Under Continuous Casting Conditions

**DOI:** 10.3390/ma18153445

**Published:** 2025-07-23

**Authors:** Fangyong Xu, Daoyao Liu, Wei Wang, Brian G. Thomas, Tianxu Wu, Kun Xu, Zhan Zhang

**Affiliations:** 1 Key Laboratory of Metallurgical Emission Reduction & Resources Recyling, Anhui University of Technology, Ministry of Education, Ma’anshan 243032, China; 2 School of Materials and Metallurgy, Wuhan University of Science and Technology, Wuhan 430081, China; 3 Department of Mechanical Engineering, Colorado School of Mines, Golden, CO 80401, USA; bgthomas@mines.edu

**Keywords:** continuous casting, microalloyed steel, high-temperature confocal laser scanning microscope, carbonitride, precipitation

## Abstract

The continuous casting of Ti-Nb microalloyed steel was simulated with high temperature confocal laser scanning microscopy (HTCLSM). Evolution of the sample surface morphology was observed in-situ, during cooling conditions chosen to represent different locations in a cast slab. Calculations with a thermodynamics model of carbonitride precipitate formation agreed with the transmission electron microscopy (TEM) analysis that fine reliefs observed on the sample surface were actually caused by interior precipitation of (Ti,Nb)(C,N). Precipitation and the resulting reliefs changed with location beneath the slab surface, simulated casting speed, and steel composition. With the same casting speed and steel composition, reliefs in the simulated slab surface sample appeared earlier and were larger than in the slab center. With increased casting speed, reliefs were observed later and decreased in size. With increased titanium or niobium content, reliefs appeared earlier and increased in number. TEM measurement showed that the precipitate diameters were mainly smaller than 4 nm, with a few between 4 and 8 nm. The property of surface reliefs observed via HTCLSM correlated qualitatively with the number and size of internal precipitates measured with TEM, showing this to be an effective tool for indirectly characterizing nanoscale secondary phase precipitation inside the sample.

## 1. Introduction

In recent years, confocal laser scanning microscopy (CLSM) has been developed as a novel and useful experimental research tool for the investigation of microstructure formation in many systems [1,2,3,4,5,6,7,8]. It has been widely used in the research of phase transformations [9,10], carbonitride precipitation [11,12] and other aspects of steel behavior, providing direct assistance in selecting steelmaking and slag materials [13], as well as in understanding and controlling the precipitate size and grain size [14]. In addition, it can help to characterize the dynamics of phase transformations, material structure, and properties [15,16]. This is also important to provide experimental benchmarks to validate computational models of precipitate formation [17].

Microalloyed steel refers to adding small amounts of carbide and/or nitride-forming elements to steel, such as Ti, Nb, V, or B. Through appropriate microalloying and heat treatment processes, microalloyed steel can achieve high strength and toughness, excellent weldability and corrosion resistance, and is widely used for bridge and building structures, pressure vessels, pipelines, and auto-body components, etc. Currently, the production of high-strength microalloyed steel by Ti and Nb additions has attracted widespread attention [18,19,20,21,22]. Nanoscale (Ti,Nb)(C,N) precipitates during the continuous casting were observed to form by diffusion in the solid steel phase depending on steel composition and cooling practice [23,24]. While causing beneficial grain refinement and secondary phase strengthening [25,26], the fine precipitates can also lead to a decrease in hot ductility and possible crack formation [27,28,29]. These carbonitrides often heterogeneously precipitated on the grain boundaries, dislocations, or pre-existing precipitate surfaces [30,31,32], and the dynamic mechanisms were quite complex. Therefore, using experimental methods to directly or indirectly observe the transformation and interaction process of secondary phase precipitation in real time is of great importance to improving the understanding of these phenomena.

Recently, a few simulations of continuous casting conditions using HTCLSM have been applied to microalloyed steel. Tang Ping et al. observed the surface morphology of the samples at different cooling rates [33,34] and proved that small “reliefs” forming on the sample surface during cooling were actually caused by the precipitation of internal carbonitride precipitates. Zou Leilei further explored and statistically analyzed the size and quantity of the appearing reliefs [35,36]. However, these studies often oversimplified the temperature history and lacked precise adjustment of steel compositions and also failed to provide a qualitative relationship between the properties of reliefs and secondary precipitates. In order to clarify these confusions, the current paper has simulated realistic temperature histories during a typical commercial continuous casting process for realistic Ti-Nb microalloyed steels, using observations in a HTCLSM. Conditions were varied to investigate the effects of slab position, casting speed, and steel composition on the formation and evolution of surface reliefs. The observations were confirmed to reveal and quantify internal precipitation behaviors using thermodynamic equilibrium precipitation calculations and TEM analysis.

## 2. Experimental Methods

### 2.1. Sample Preparation

Ultra-low carbon steel (labeled S0 here) containing 99.8% Fe was used as the base material, and different amounts of ferrotitanium, ferroniobium, carbon powder, and aluminum foil were added to finely adjust the steel composition into 3 microalloyed steel samples. The samples were fully melted using a vacuum induction furnace (Jinzhou Mait Vacuum Equipment Co., Ltd., Jinzhou, China), electromagnetically stirred for another 15 min at a power of 12 kW, and then water-cooled and cast into 1-kg ingots, resulting in samples S1 containing about 0.025 wt.% niobium, S2 containing about 0.05 wt.% niobium, and S3 containing about 0.05 wt.% niobium and 0.012 wt.% titanium. The specific compositions of the four samples were measured by the spark optical emission spectrometer (Thermo Fisher Scientific, Waltham, MA, USA) and oxygen-nitrogen-hydrogen analyzer (Laboratory Equipment Corporation, St. Joseph, MI, USA), and given in Table 1.

### 2.2. Confocal Laser Scanning Microscopy Measurements

The as-cast ingots were cut with a wire-electrode into cylindrical test specimens with a diameter of 7 mm and a height of 3 mm, cleaned, and polished. A HTCLSM VL2000Dx-SVF17SP (Japan Yonekura Mfg. Co., Ltd., Yokohama, Japan) with protective argon surroundings was used to apply realistic temperature histories to the specimens to simulate the commercial continuous casting process, and real-time videos of microstructure evolution at the sample surface were recorded at 3 frames per second. Further information on the CLSM is provided elsewhere [37].

**Figure 1:**
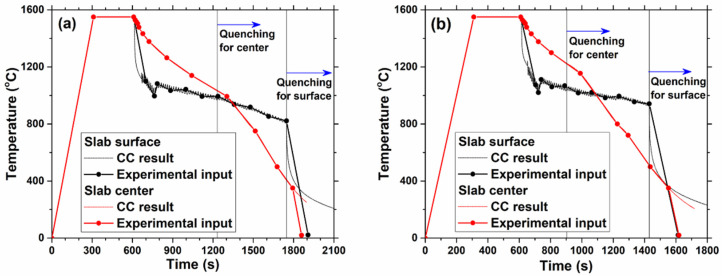
Temperature curves for experiments to simulate cooling during continuous casting at a (**a**) casting speed of 0.9 m/min and (**b**) 1.25 m/min.

Each sample was first heated at a maximum rate of 5 °C/s to 1550 °C (roughly 20 °C above the liquidus temperature), and the liquid was kept at 1550 °C for 5 min to fully dissolve the alloy. Then, according to results from the heat transfer model CON1D [38], typical temperature histories at the slab surface and center during the complete continuous casting process were calculated and applied to the samples. The cooling histories shown in Figure 1 correspond to the surface and center of a 152 mm-thick thin-slab casting [39] at casting speeds of 0.9 m/min and 1.25 m/min. Because the number of temperature steps that can be set in the HTCLSM experiment was restricted to a maximum of 16, the actual experimental temperature curves are also shown in Figure 1 for comparison. For the slab center samples, the holding time at 1550 °C was partly shortened to reduce high-temperature damage to the instrument.

Comparing the temperature curves, it is seen that higher continuous casting speed caused faster cooling. The slab surface temperature oscillated significantly in the secondary cooling spray zone, as the slab passed by individual rolls and spray nozzles, while the slab center temperature decreased continuously after final solidification at the metallurgical length. Note that the amount of water sprayed during secondary cooling, which was set to increase linearly with casting speed, was found to be insufficient to compensate for the resulting decrease in cooling time, so the slab surface temperature was slightly higher at the higher casting speed. The slab center temperature also increased with higher casting speed. 

## 3. Thermodynamic Model Calculation of Equilibrium Precipitation

**Figure 2:**
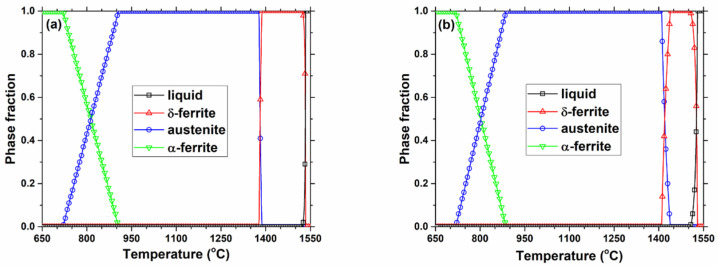

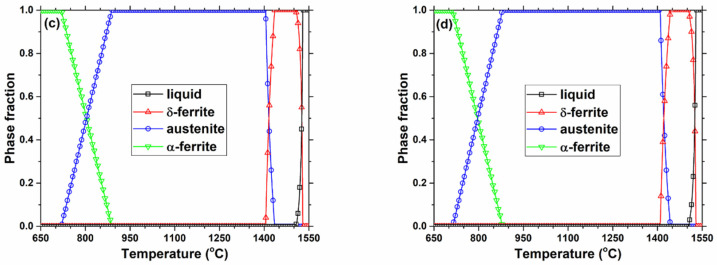
Evolution of steel phases with temperature for the studied grades (**a**) S0, (**b**) S1, (**c**) S2, (**d**) and S3.

According to the steel compositions in Table 1, the equilibrium steel phases were calculated using the CON1D model [38], and shown in Figure 2. As temperature decreased, all steel grades followed a similar path of phase transitions from liquid to δ-ferrite to austenite to α-ferrite (and Fe_3_C). As the carbon content of the steel increased, the liquidus and solidus solidification temperatures decreased, the transition temperature from δ-ferrite to austenite increased, and the transition temperature from austenite to alpha ferrite decreased. The effects of titanium and niobium alloying on the steel phases were relatively insignificant.

**Figure 3:**
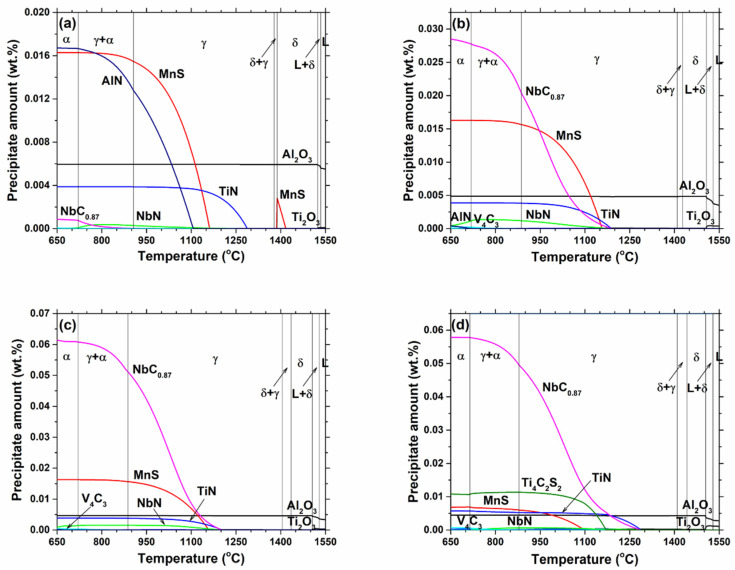
Equilibrium precipitate phases vs. temperature for the studied grades (**a**) S0, (**b**) S1, (**c**) S2, and (**d**) S3.

Next, the steel compositions in Table 1 and corresponding phase fractions in Figure 2 were input into the equilibrium thermodynamic precipitation model, EQPrecip [40], to calculate the types and amounts of precipitated phases at equilibrium. The results, shown in Figure 3, included carbonitride precipitates, TiN, TiC, NbN, NbC_0.87_, VN, and V_4_C_3_, which were considered to be a single mutually-soluble precipitate phase group (Ti,Nb,V)(C,N) [40,41,42,43]. For all steel grades, most of the aluminum and some of the titanium formed stable Al_2_O_3_ and Ti_2_O_3_ inclusions in the liquid steel. Next, carbonitrides of titanium, niobium, and vanadium formed sequentially with decreasing temperature, as their solubilities decreased. The titanium nitride and carbide precipitates generally formed first at higher temperatures in all alloys, in spite of the low Ti contents, owing to the smallest solubility of the titanium carbonitride. 

For the ultra-low carbon steel grade S0, MnS began to form in δ-ferrite but completely redissolved in austenite due to its increased solubility in that steel phase. As temperature decreased, TiN, MnS, and AlN formed in succession. A very small amount of NbN began to form at 1050 °C, and later, part of its niobium was consumed by NbC_0.87_ formation, resulting in decreasing NbN below 750 °C. For the S1 and S2 grades, which had higher carbon and niobium contents, the NbN and NbC_0.87_ precipitation began at higher temperatures and grew to form higher amounts during cooling. Furthermore, a small quantity of V_4_C_3_ started to form at lower temperatures. For the S3 grade, the higher titanium content caused TiN to precipitate first in the austenite and increased the solid solution temperature of (Ti,Nb,V)(C,N).

## 4. Results

### 4.1. General In-Situ Observations of High-Temperature Confocal Laser Scanning Microscope

**Figure 4:**
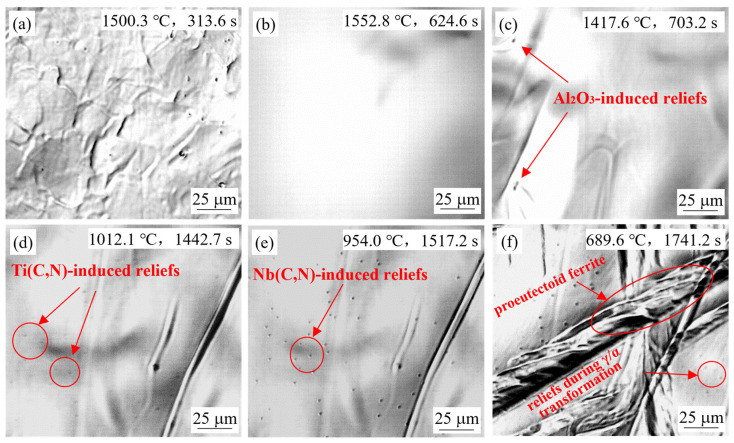
In-situ observation of S2 steel sample for simulated cooling at slab center at 0.9 m/min.

Cooling experiments with simulated continuous-casting conditions were performed in the HTCLSM for steel grades S1, S2, and S3. Extensive bulging of the surface generating reliefs was observed at four times during the entire cooling process. Example results were shown in Figure 4 for in-situ observations of steel S2 at the simulated slab center with the casting speed of 0.9 m/min. After heating to 1500 °C, there were still some surface reliefs remaining in Figure 4a. After heating to 1550 °C and holding for 5 min, the sample was completely melted, and the surface reliefs had completely disappeared. 

During cooling, when the temperature dropped to 1450–1360 °C, a few reliefs first appeared, as seen in Figure 4c. This was likely caused by unmelted alumina particles floating near the sample surface or lattice distortion generated by alumina particles forming inside the sample just after solidification. This was confirmed by the fact that the formation temperature of alumina was higher than the solidification temperature of steel. The subsequent two bulging phenomena, as shown in Figure 4d,e, mainly occurred in the temperature range of 1100 °C–900 °C, which was believed to be caused by the precipitation of new carbonitrides within the austenite. With decreasing temperature, the originally stable solute atoms underwent segregation and initialized the secondary precipitation. This process caused the local area around the secondary precipitates to become depleted of atoms, forming a different specific volume with the nearby matrix, making the volume of this area expand, and resulting in the formation of reliefs on the sample surface [33,34,35,36]. During the final quenching stage, when the sample was cooled to 750 °C–650 °C, reliefs were observed again. During this time, the sample was in the transition temperature range from austenite to α-ferrite. With the decreasing solubility of carbonitrides and the increasing diffusion coefficient of alloying elements, tiny carbonitrides rapidly precipitated out along the γ/α interface or from the ferrite. Accordingly, proeutectoid ferrite and new finer reliefs were observed, as shown in Figure 4f. By changing casting conditions and steel compositions, the appearance times, temperatures, and patterns of the observed reliefs during the mid-term continuous casting process were quite different. The details are carefully discussed in the next sections.

#### 4.1.1. Effect of Slab Position

Figure 4d,e show the in-situ observed results of the simulated slab center for steel S2 at a casting speed of 0.9 m/min. Small reliefs appeared at 1012.1 °C for the second time, and these reliefs gradually became larger and clearer. When the temperature further decreased to 954.0 °C, more reliefs appeared on the sample surface and increased in number and size as the cooling continued. From a thermodynamic perspective, the reliefs in Figure 4d can be attributed to titanium-rich carbonitride that has completely precipitated at a relatively higher temperature, while the reliefs in Figure 4e were induced by niobium-rich carbonitride that only fully precipitated at a relatively lower temperature range. Figure 3c shows that the solid solution temperature of (Ti,Nb,V)(C,N) for steel S2 was 1204 °C, but the true dynamic precipitation behavior was governed by the combined kinetic effects, including supersaturation and diffusion rate. In the high-temperature region, the diffusion rate was high, but supersaturation was low, while in the low-temperature region, the supersaturation was high, but the diffusion rate was low. Therefore, the fastest precipitation rate was obtained at the “nose” in the intermediate temperature region [44,45]. Both HTCLSM experimental observations and kinetic model calculations of isothermal precipitation confirmed that the “nose point” temperature of steel S2 was around 1000 °C [46], which was close to the observed appearance temperature of the reliefs in Figure 4d. 

**Figure 5:**
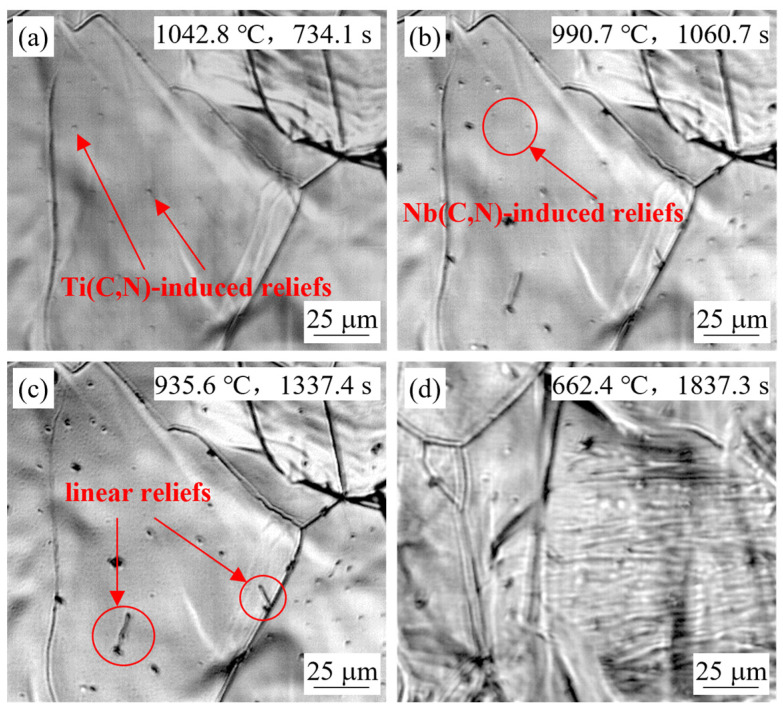
In-situ observation of S2 steel sample for simulation of slab surface at 0.9 m/min.

Figure 5 shows results observed on the slab surface sample for steel S2 at the same casting speed. During cooling, relief bulging was observed two times at temperatures of 1042 °C and 990.7 °C, respectively. These temperatures were slightly higher than those when the reliefs appeared on the slab center sample, but the corresponding times were much earlier. This was because the slab surface was cooled much earlier, making it experience a lower temperature range first. In the early stage of the secondary cooling, carbonitrides began to form on the slab surface, resulting in the formation of reliefs. The slab center remained at high temperature throughout most of the casting process, so the carbonitrides formed towards the end of the casting or during the later rapid quenching stage, when the reliefs were observed.

#### 4.1.2. Effect of Casting Speed

**Figure 6:**
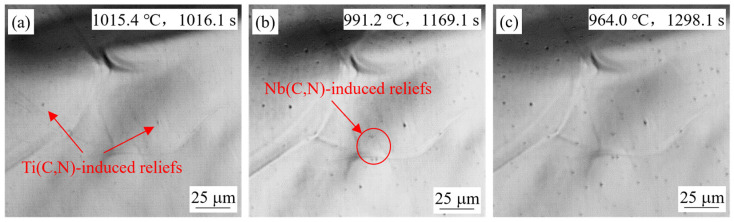
In-situ observation of S2 steel sample for simulation of slab surface at 1.25 m/min.

Observations of the simulated slab surface samples for steel S2 at different casting speeds are compared in Figure 5 and Figure 6. At a casting speed of 1.25 m/min, the temperatures at which two bulging reliefs appeared were 1015.4 °C and 991.2 °C, respectively, which were close to the temperatures at which the reliefs appeared at the casting speed of 0.9 m/min. Although the slab surface sample was cooled later, the reliefs appeared earlier at 0.9 m/min. This was because the slab surface stayed in the low-temperature region longer with the lower casting speed. From a kinetic perspective, precipitation required not only reaching the precipitation temperature but also some holding time, so the reliefs appeared sooner for the 0.9 m/min sample. The total cooling time experienced at the higher casting speed was shorter, resulting in a smaller relief size distribution on the final morphology of the sample surface.

#### 4.1.3. Effect of Steel Composition

**Figure 7:**
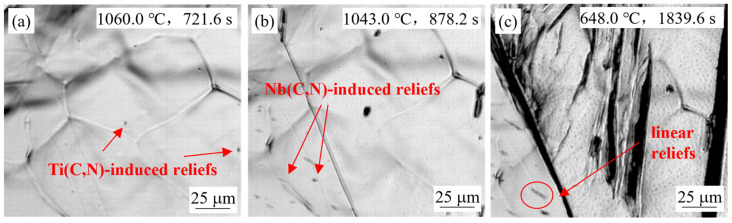
In-situ observation of S3 steel sample for simulation of slab surface at 0.9 m/min.

**Figure 8:**
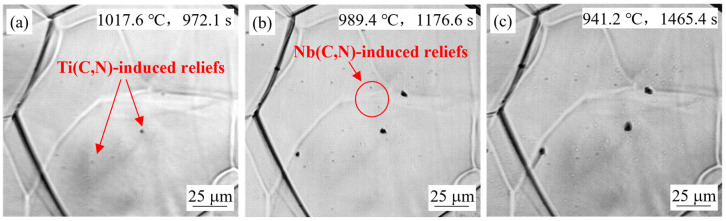
In-situ observation of S1 steel sample for simulation of slab surface at 0.9 m/min.

Figure 5 and Figure 7 compare the in-situ observations of the simulated slab surface samples for steel S2 and S3 at a casting speed of 0.9 m/min. Relief bulging phenomena appeared two times at 1060 °C and 1043 °C for steel S3, with temperatures and times consistently higher and earlier than those for steel S2. This was due to the lower solubility of TiN. Meanwhile, a small number of linearly-distributed reliefs can be seen in Figure 5c and Figure 7c, which were likely caused by carbonitride precipitation on dislocations or grain boundaries. Figure 8 shows the simulated slab surface sample of steel S1 at the same casting speed, and bulging phenomena appeared two times at 1017.6 °C and 989.4 °C. Compared with the results of steel S2, the temperatures of relief formation were almost the same, but the times were a little later, due to the niobium content being reduced by half. The number of reliefs observed for steel S2 was lower, indicating that more reliefs appeared with increasing niobium content. Moreover, an obvious size difference between the earlier Ti(C,N)-induced reliefs and later Nb(C,N)-induced reliefs can be clearly seen in Figure 5b, Figure 6b, Figure 7b and Figure 8b, which indirectly indicated the effect of titanium and niobium microalloying on the secondary precipitation.

**Figure 9:**
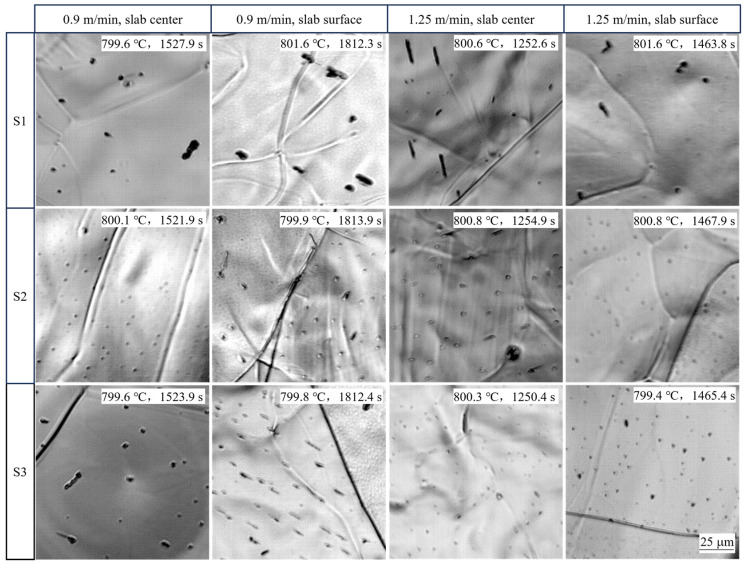
Comparison of in-situ observations of samples at 800 °C after simulated cooling of slab center and surface samples at two casting speeds for three steel grades.

A summary of the HTCLSM in-situ observations at 800 °C is presented in Figure 9, which compares the observed microstructures at the same magnification for both casting speeds and all three steel compositions, both at the slab surface and center. Comparing between the rows, steel S1 was seen to have the fewest and largest reliefs, indicating that increasing either niobium or titanium content generated more fine reliefs. Comparing between the columns, the simulated cooling history at both the lower casting speed and at the slab surface had larger reliefs. All of these results can be explained well by classical precipitation kinetics theory, assuming that the number and size of the observed reliefs correlate proportionally with the number and size of the internal precipitates.

### 4.2. Characterization by Transmission Electron Microscopy

To measure the actual size distribution of the precipitates, the samples obtained after the HTCLSM experiment were sliced and subjected to double jet ion thinning technology [47] of Gatan 691 (Gatan, Inc., Pleasanton, CA, USA) for observation using a Jeol JEM-2100 (JEOL Ltd., Tokyo, Japan) transmission electron microscope. Using multiple ion thinning passes to create 3 mm-diameter circular disks which have a small drilled hole in the center and the thinnest areas of only a few tens of nanometers thick, and careful position adjustment, the precipitates were clearly observed with TEM. Compared with the traditional carbon extraction replica techniques [48,49], direct ion thinning enabled observation of a larger area and avoided incomplete extraction efficiency, so more accurate statistics of secondary precipitate particles were expected.

**Figure 10:**
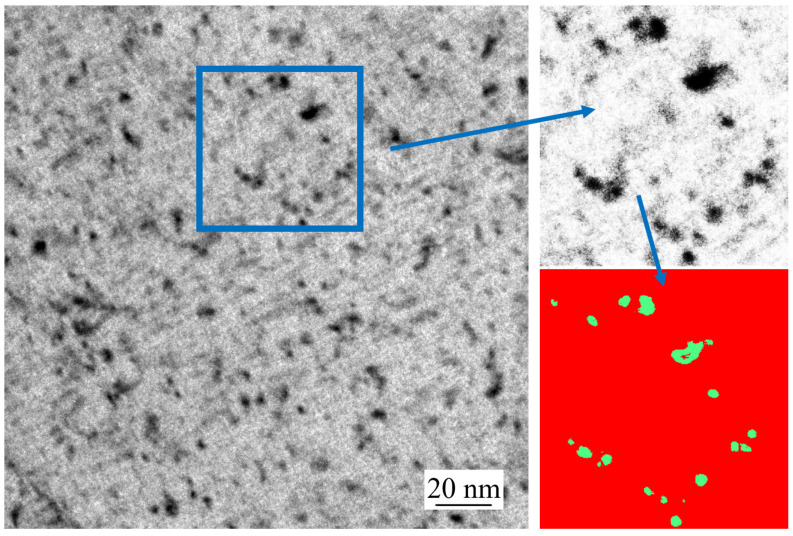
TEM characterization of an S2 steel sample for simulation of the slab surface at 0.9 m/min.

**Figure 11:**
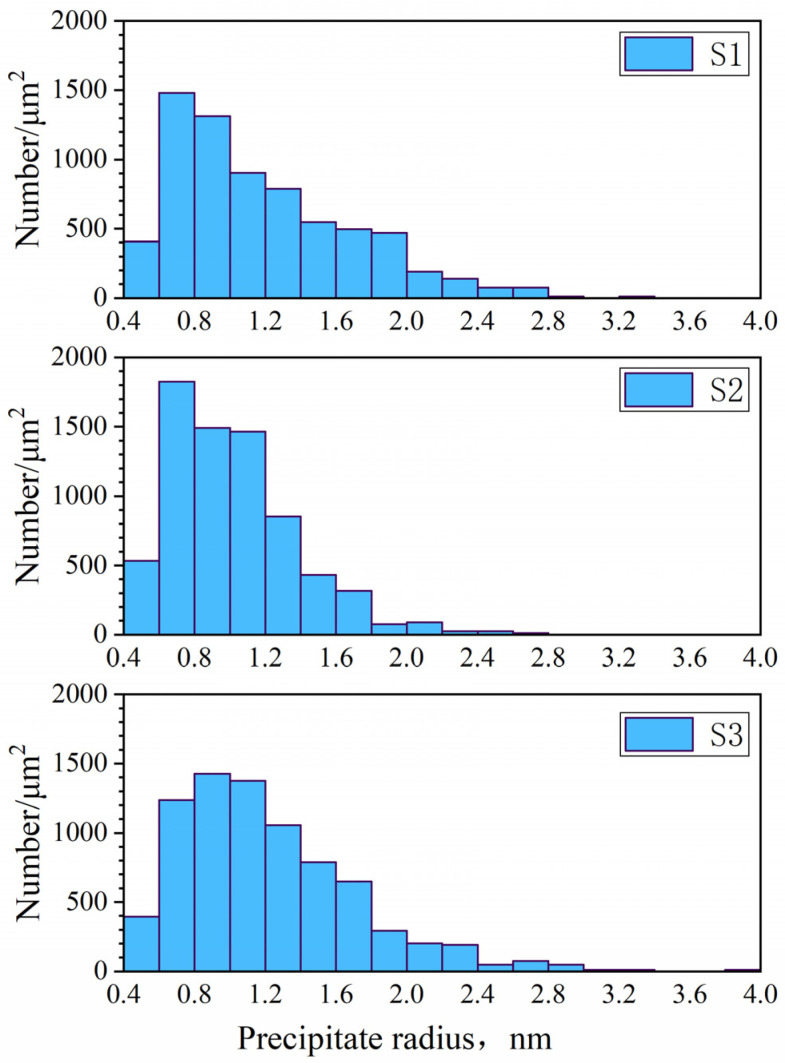
Precipitate size distributions measured by TEM in three steel grades for simulation of the slab surface at 0.9 m/min.

The TEM images were imported into the software Fiji ImageJ 2.16.0 with the Weka segmentation plugin and the precipitate particles were statistically analyzed. An example image is shown in Figure 10. Assuming that each precipitate was perfectly circular, the visualized precipitate area was converted into an equivalent average precipitate radius. The size distributions of precipitates are shown in Figure 11 on the slab surface for three steels at the casting speed of 0.9 m/min. Due to the resolution limit of TEM measurement, only shaded areas with an average radius greater than 0.4 nm were counted. It can be seen that the precipitate radii of all steel grades are mostly smaller than 2 nm, with a few distributed between 2 and 4 nm. The precipitates in steel S2 are smaller than those of S1. As the niobium content increases, the supersaturation increases, resulting in a higher nucleation rate and smaller critical nucleus size. Therefore, the number density of precipitates has increased, while their radii decreased [50,51].

Unlike steel S1, the size distribution of precipitates of steel S2 exhibited slightly bimodal characteristics, while this bimodal size distribution was even more obvious for steel S3. The peak at ~2.7 nm corresponds to titanium-rich carbonitrides, as their low solubility promotes full precipitation at higher temperatures. The peak at ~0.9 nm corresponds to niobium-rich carbonitrides formed at relatively lower temperatures, when the niobium gradually reacted with the residual carbon and nitrogen to heterogeneously precipitate on the surfaces of existing precipitated Ti(C,N) particles, while also possibly precipitating homogeneously from the matrix.

**Figure 12:**
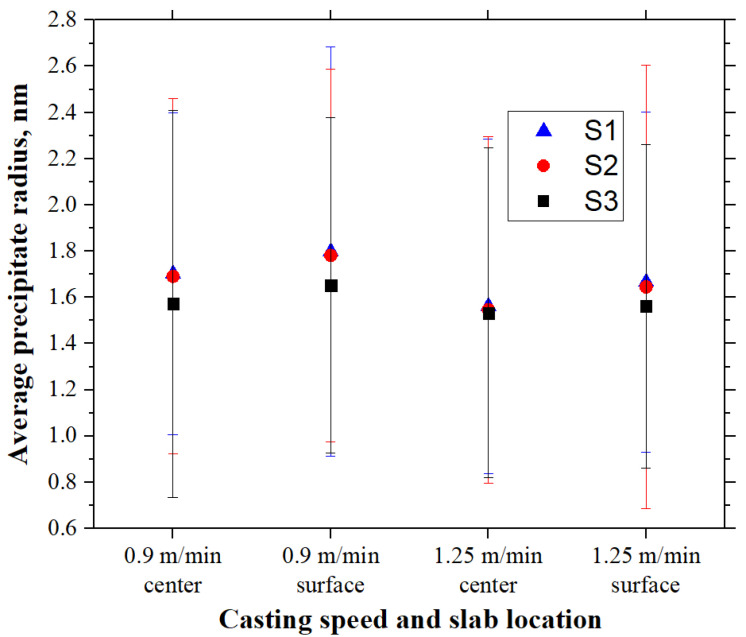
Average precipitate radii in three steel grades for simulations of the slab surface and center at two casting speeds.

A comparison of the average precipitate radii of three steel compositions for simulated cooling conditions on the slab surface and center at two casting speeds (0.9 and 1.25 m/min) is shown in Figure 12. The average precipitate radius on the slab surface was found to be larger than that found at the slab center, and the average precipitate radius was larger at the lower casting speed for all 3 steels (S1, S2, and S3). The average precipitate radii in steels S1 and S2 are similar and generally greater than those in steel S3. This indicates that increasing niobium content has little effect on the carbonitride size, but adding a tiny amount of titanium can effectively refine the precipitate sizes. For the slab center at the casting speed of 1.25 m/min, precipitation only occurs during the final quenching stage, so the influence of steel composition on average radii is small.

## 5. Discussion

**Figure 13:**
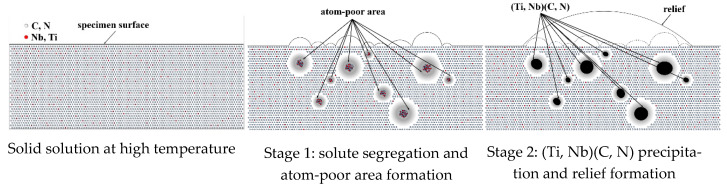
Illustration of surface relief formation.

The observations of relief morphologies in the HTCLSM experiments showed a strong correlation with the number densities and sizes of the precipitates in the TEM measurements. This suggested that more and larger reliefs corresponded to a larger number of precipitates and to larger precipitate sizes. The mechanism was illustrated in Figure 13, as explained in previous studies [33,34,35,36].

Subsurface precipitates push up the surface, creating “reliefs” in proportion to their local size and number (stage 1). As the interior precipitates coalesce and grow in size, the reliefs become larger and fewer in number as well (stage 2). The reliefs are visible as dark regions in the micrographs. Although the reliefs are very much fewer and larger (Figure 13 is not drawn to exact scale), the trends of the number and size evolution of the reliefs correlate directly and qualitatively with those of the interior precipitates. Thus, if care is taken, based on a fundamental understanding of the precipitation mechanisms, then the generation of reliefs on the specimen surface can be used to indirectly characterize the internal secondary phase precipitation.

## 6. Conclusions

Samples of several microalloyed Ti-Nb steels were cooled under conditions to simulate those in commercial continuous casting at different casting speeds and locations (slab center and surface). In-situ observations of the sample surfaces were carried out using a HTCLSM, and TEM was performed to measure precipitate size distributions on the finally-quenched samples. Precipitation calculations with an equilibrium thermodynamic model matched well with the observed reliefs and TEM measurements. Specific conclusions are as follows:

(1) Small reliefs observed on the sample surface during HTCLSM were confirmed to result from internal precipitation of (Ti,Nb)(C,N). These reliefs appeared sequentially as temperature decreased during cooling. The formation of reliefs depended on the simulated slab position, the casting speed, and the steel composition. The reliefs formed earlier on the slab surface than in the slab center due to earlier cooling, and their size was larger. The reliefs appeared later with increasing casting speed, and their size was smaller. When the titanium or niobium content in the steel increased, the surface reliefs appeared earlier, and their number increased.
(2) TEM measurements showed that the precipitate radii of the simulated continuous casting experiments were mostly distributed within 2 nm, with a few larger precipitates at 2–4 nm. The average radius of precipitates decreased with increasing distance from the casting surface, casting speed, or titanium or niobium content in the steel sample.
(3) The surface relief morphologies observed by HTCLSM showed a strong positive qualitative correlation with the number and sizes of precipitates measured by TEM, although the TEM precipitate size distributions showed much larger numbers of much smaller particles. Therefore, the relatively easy in-situ observation of micron-scale surface reliefs using HTCLSM was demonstrated to be an effective tool for indirect characterization of nanoscale secondary phase precipitation inside steel samples.
(4) The experimental measurements presented here can serve as a benchmark for future modeling studies to predict starting times and precipitate size distributions of (Ti,Nb)(C,N) precipitation.

## Figures and Tables

**Table 1:** Chemical compositions of experimental steels (mass fraction).

	C	O	N	Nb	Ti	V	Si	Mn	Al	S	P	Ni	Cr
S0	0.004	0.0029	0.0066	0.0008	0.003	0.0005	0.018	0.073	0.027	0.006	0.009	0.017	0.044
S1	0.048	0.0023	0.0011	0.026	0.003	0.0004	0.032	0.070	0.006	0.006	0.010	0.17	0.079
S2	0.047	0.0022	0.0011	0.056	0.003	0.0005	0.032	0.071	0.006	0.006	0.010	0.15	0.076
S3	0.053	0.0021	0.0013	0.052	0.012	0.0007	0.032	0.071	0.006	0.005	0.010	0.20	0.087

## Data Availability

The original contributions presented in this study are included in the article. Further inquiries can be directed to the corresponding authors.
